# Emerging innovative treatment strategies for advanced clear cell renal cell carcinoma

**DOI:** 10.1093/oncolo/oyae276

**Published:** 2024-10-14

**Authors:** Sharon H Choi, Yu-Wei Chen, Justine Panian, Kit Yuen, Rana R McKay

**Affiliations:** Division of Hematology Oncology, University of California San Diego, San Diego, CA, United States; Division of Hematology Oncology, University of California San Diego, San Diego, CA, United States; Division of Hematology Oncology, University of California San Diego, San Diego, CA, United States; Department of Urology, University of California San Diego, San Diego, CA, United States; Division of Hematology Oncology, University of California San Diego, San Diego, CA, United States; Department of Urology, University of California San Diego, San Diego, CA, United States

**Keywords:** renal cell carcinoma, treatment options, antiangeiogenic, immune checkpoint inhibitors

## Abstract

Dramatic advances in biological discoveries, since the 1990s, have continued to reshape the treatment paradigm of metastatic renal cell carcinoma (RCC). Von Hippel Lindau (VHL) gene alterations are associated with pro-angiogenic activity and are central to the pathogenesis of clear cell RCC (ccRCC), the most predominant histologic subtype of RCC. Antiangiogenic strategies revolving around this VHL/HIF/VEGF axis have been shown to improve survival in metastatic ccRCC. The discovery of immune checkpoints and agents that target their inhibition introduced a new treatment paradigm for patients with RCC. While initially approved as monotherapy, studies investigating immune checkpoint inhibitor combinations have led to their approval as the new standard of care, providing durable responses and unprecedented improvements in clinical outcome. Despite these advances, the projected 14 390 deaths in 2024 from RCC underscore the need to continue efforts in expanding and optimizing treatment options for patients with metastatic RCC. This article reviews key findings that have transformed the way we understand and treat metastatic RCC, in addition to highlighting novel treatment strategies that are currently under development.

## Teaser

This article reviews key findings that have transformed the way we understand and treat metastatic renal cell carcinoma and highlights novel treatment strategies that are currently under development.

**Implications for practice**

Biological discoveries in renal cell carcinoma (RCC) have translated into the development of multiple treatment options. Over the past 3 decades, the treatment paradigm has dramatically shifted with the introduction of immune checkpoint inhibitors (ICI), now the cornerstone of metastatic RCC management either alone or in combination with targeted agents. Although these advances have revolutionized treatment options for metastatic RCC, questions remain on how to optimally approach management of relapsed or refractory disease. This article will explore the pivotal findings regarding the pathogenesis of metastatic renal cell carcinoma and how these discoveries have influenced novel treatments for this disease.

## Introduction

Renal cell carcinoma (RCC) is a common cancer in the United States, with estimates of over 81 000 new diagnoses in the year 2024.^1^ The vast majority (approximately 75%-80%) of patients with RCC are diagnosed with the clear cell (ccRCC) subtype.^2^ Most patients present with localized disease amenable to definitive nephrectomy, however, between 20% and 40% of patients have been shown to develop metastatic recurrence despite curative-intent local therapy.^3^ Additionally, approximately 20%-30% of patients present with advanced or metastatic disease at the time of initial diagnosis.^4^

**Figure 1.**
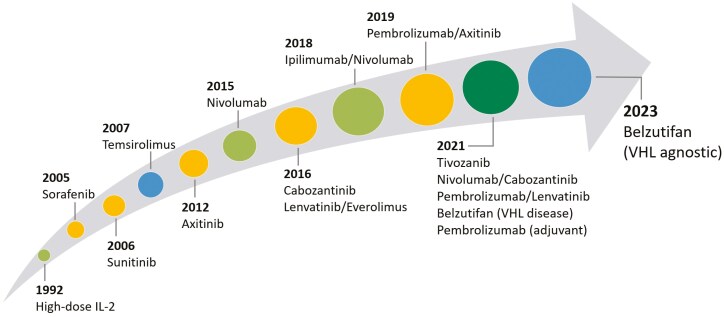
Timeline of FDA approved agents for the treatment of metastatic renal cell carcinoma.

Over the last 2 decades, the management of metastatic ccRCC has evolved from cytokine-based therapy such as interferon-alpha and interleukein-2 (IL-2) to the development of targeted approaches. In 1992, the Food and Drug Administration (FDA) approved the use of high-dose IL-2 for patients with metastatic RCC, becoming the first immunotherapeutic agent approved for the treatment of cancer.^5,6^ Although high-dose IL-2 provided durable survival benefit in a small subset of patients with metastatic RCC,^7^ response rates were limited until the development of targeted agents. In 2005 and 2006, sorafenib and sunitinib became the first tyrosine kinase inhibitor (TKI) agents approved for targeting the vascular endothelial growth factor (VEGF) receptor,^8,9^ followed by subsequent VEGF and mTOR treatment therapy strategies in the refractory setting (Figure 1). Since 2018, the treatment paradigm has evolved with the introduction and approval of immune checkpoint inhibitor (ICI) combination therapies for first-line treatment of metastatic ccRCC. The advancements in translational discoveries and treatment options are manifested by the improvement in median survival for patients with metastatic ccRCC—from less than a year in the early 1990s to nearly 5 years in some recent trials.^10,11^ Despite these marked improvements in survival, it is estimated that over 14 000 patients in the United States. will succumb to their disease in 2024,^1^ highlighting the need for novel and effective options in the treatment-refractory setting. In this article, we aim to summarize the evolving evidence that has shaped current management, and to review emerging novel therapeutics that are currently under investigation for the treatment of metastatic ccRCC.

### Antiangiogenic agents

#### Hypoxia-inducible factor (HIF) inhibitors

The considerable progress in understanding the pathogenesis of RCC, along with the central role of the VHL/HIF/VEGF axis, have provided a foundation for the development of targeted therapies for advanced ccRCC.^12-14^ Under normal conditions, VHL serves as a tumor suppressor protein that has been shown to inversely regulate transcriptions factors, HIF-1α and HIF-2α, through ubiquitin-mediated proteasomal degradation, thereby preventing angiogenesis and tumor growth. In VHL-altered RCC, HIF-2α is constitutively activated, stimulates erythropoietin expression and induces pro-angiogenic transcription factors such as VEGF, PDGF, and TGF-α. The loss of VHL activity is estimated to occur in approximately 90% of ccRCC and thought to be an early event in the tumorigenesis of ccRCC.^15,16^

Belzutifan, the first-in-class HIF-2α inhibitor, was approved by the US Food and Drug Administration (FDA) in 2021 for the treatment of RCC with germline *VHL* mutations (VHL disease). This approval was based on the results of a phase 2 trial, which showed an overall response rate (ORR) of 49% in RCC patients with VHL disease.^17^ However, the efficacy of HIF-2α inhibition in non-familial RCC remained relevant, considering that approximately 90% of individuals with ccRCC harbor somatic *VHL* mutations. Accordingly, multiple trials have evaluated the use of belzutifan for pretreated advanced ccRCC. Results from LITESPARK-001 showed an objective response in 14 of the 55 patients (25%) with a median progression-free survival (PFS) of 14.5 months (95% CI, 7.3-22.1) in the treatment refractory setting with belzutifan monotherapy. The phase 3 LITESPARK-005 trial compared single-agent belzutifan to everolimus in patients with advanced ccRCC whose disease progressed following both an ICI and a VEGF-TKI.^18^ The results demonstrated a PFS benefit in patients who received belzutifan compared to everolimus (21 vs 17.2 months, HR 0.75, 95% CI, 0.63-0.90) without new or unexpected safety signals for belzutifan. On the basis of these safety and efficacy endpoints, in December 2023, the FDA approved the use of belzutifan for pretreated patients with advanced ccRCC.

The first trial to report the antitumor activity of combination HIF-2α therapy was LITESPARK-003, a single-arm phase 2 study, that examined belzutifan plus cabozantinib in untreated (cohort 1) and previously treated advanced ccRCC (cohort 2). The results showed that the combination of belzutifan and cabozantinib in the untreated population (cohort 1) provided an ORR of 70% with a median duration of response (DOR) of 28.6 months across IMDC risk categories.^19^ The ORR in pretreated ccRCC patients was 31%. Given the single arm design, the contribution of the component parts was not defined, however, the cabozantinib control arm of the CONTACT-03 trial can be utilized as a reference standard of cabozantinib later-line activity in the modern era (ORR 41%).^20^ The results from LITESPARK-003 provide rationale for further studies, such as the ongoing phase 3 LITESPARK-011 trial, which seeks to demonstrate the efficacy of belzutifan plus lenvatinib, a VEGF-TKI, for pretreated advanced ccRCC.^21^ Additionally, a phase 3 study is comparing the addition of belzutifan to pembrolizumab/lenvatinib with pembrolizumab/lenvatinib for first-line advanced ccRCC.^22^ There are several active trials that are currently investigating the clinical benefit of belzutifan in combination with other agents in the adjuvant and advanced settings (Table 1).

**Table 1.** Ongoing clinical trials of HIF-2α inhibitors in advanced RCC.

Treatment	Setting	Phase	Primary endpoints	Identifier
Pembrolizumab +/- belzutifan	Adjuvant therapy of ccRCC	3	DFS	NCT05239728
Favezelimab (anti-LAG-3)/pembrolizumab + lenvatinib or Vibostolimab (anti-TIGIT)/pembrolizumab + belzutifan vs. pembrolizumab + lenvatinib	1L advanced ccRCC	1/2	Lead-in phase: DLT; AEs Efficacy phase: DLT; AEs; ORR	NCT04626479
Pembrolizumab + belzutifan + lenvatinib or pembrolizumab/quavonlimab (anti-CTLA-4) + lenvatinib vs. pembrolizumab + lenvatinib	1L advanced ccRCC	3	PFS/OS	NCT04736706
HC-7366 (GCN2 activator) + belzutifan	3L + advanced ccRCC	1	MTD, RP2D	NCT06234605
Belzutifan (standard dose vs high dose)	3L + advanced ccRCC	2	ORR	NCT04489771
Belzutifan + lenvatinib vs cabozantinib	2L + advanced ccRCC after ICI	3	PFS/OS	NCT04586231
NKT2152 (selective HIF-2α inhibitor)	2L + advanced ccRCC	1/2	Phase 1: DLT; RP2D Phase 2: ORR	NCT0511933

Abbreviations: 1L, first line; 2L+, second-line or later; 3L+, third-line or later; AEs, adverse events; ccRCC, clear cell renal cell carcinoma; DFS, disease-free survival; DLT, dose-limiting toxicity; ICI, immune checkpoint inhibitor; ORR, overall response rate; OS, overall survival; PFS, progression-free survival; RP2D, recommended phase 2 dose..

#### Tyrosine kinase inhibitors

In 2005 and 2006, sorafenib and sunitinib became the first FDA-approved TKI therapies targeting the VEGF receptor based on evidence from phase 3 trials.^8,9^ The majority of TKI therapies for RCC target the VEGF receptor, a downstream component of the VHL/HIFα pathway, which promotes angiogenesis and tumor proliferation. Other VEGF TKIs approved for the treatment of advanced RCC include pazopanib, axitinib, and cabozantinib.^23-25^ These agents served as first-line therapies until 2018, when ICI-based combination regimens became the standard of care for frontline treatment of metastatic RCC, with patients either receiving dual ICI or ICI/TKI therapies at diagnosis.^26-29^

An emerging strategy in the development of TKIs includes targeting AXL, a member of the TAM (TYRO3, AXL, MER) family of receptor tyrosine kinases, which is regulated by the VHL/HIF-α axis and associated with aggressive tumor behavior and poor prognosis.^30,31^ The accumulation of HIF-2α in ccRCC consequent to VHL loss has been shown to induce AXL expression, which plays an important role in RCC pathogenesis, metastasis, and tumor invasion.^31^ AXL has also been associated with an epithelial-mesenchymal phenotype and an immunosuppressed tumor microenvironment.^32^ Additionally, VEGF-targeted TKI therapy has been shown to induce AXL upregulation, leading to TKI resistance.^33^ Taken together, these findings provide rationale in identifying AXL signaling pathway as a therapeutic target to abrogate tumor progression in advanced ccRCC. In 2022, the FDA granted fast track designation to batiraxcept, a competitive inhibitor of the AXL signaling pathway, for the treatment of refractory advanced RCC.^34^ The FDA designation was based on phase 1b results, which showed that batiraxcept plus cabozantinib in pretreated advanced ccRCC demonstrated an ORR of 42%, median PFS of 9.3 months, and a favorable safety profile.^35^ The phase 2 study included 3 cohorts: batiraxcept monotherapy in heavily pretreated patients with limited subsequent-line treatment options; batiraxcept plus cabozantinib with at least 1 prior therapy; and batiraxcept plus cabozantinib with nivolumab in the first-line setting. The results demonstrated antitumor activity in patients who received first-line batiraxcept plus cabozantinib with nivolumab (ORR 55%) and second- or later-line batiraxcept plus cabozantinib (ORR 36%).^36^ Single-agent batiraxcept exhibited minimal clinical activity in the refractory setting (ORR 0%). Additional AXL inhibitors include PF-07265807 (ARRY-067), a small-molecule inhibitor of both AXL and MER kinases, that is currently in phase 1 development in combination with ICI and a VEGF TKI for the treatment of advanced ccRCC (NCT04458259).

### Emerging immune checkpoint inhibitors

#### CTLA-4 inhibitor and ICI/probiotic combinations

Cytotoxic T lymphocyte antigen-4 (CTLA-4) was the first identified immune checkpoint molecule and is expressed on the surface of cytotoxic CD8+ T cells.^37^ CTLA-4 was later described as a negative regulator of T-cell activation by competing with CD28 receptor for B7 ligand binding on antigen presenting cells, an essential costimulatory signal for T-cell activation and expansion.^38,39^ CTLA-4 was subsequently recognized to be found on regulatory (Treg) cells to sustain its suppressive activity by sequestering B7 binding, thereby abrogating costimulatory signaling in other cytotoxic T cells.^40,41^ Treatment strategies targeting the CTLA-4 and PD-1 axes in metastatic RCC have been pivotal in changing the treatment landscape for advanced and metastatic ccRCC.^27,42^ The current standard first-line therapy for advanced ccRCC includes an immune checkpoint inhibitor doublet of ipilimumab (anti-CTLA-4) and nivolumab (anti-PD-1) based on the results from CheckMate 214.^43^ CBM588, a live probiotic containing *Clostridium butyricum*, was evaluated in combination with nivolumab and ipilimumab in previously untreated metastatic RCC in a phase 1 trial in which the bacterial combination elicited an ORR of 58% compared with 20% for patients who received nivolumab/ipilimumab alone.^44^ In another phase 1 study, patients with untreated metastatic RCC who received cabozantinib/nivolumab combined with CBM588 had a higher ORR compared to patients who received cabozantinib/nivolumab alone (74% vs 20%).^45^ The promising outcomes from these 2 early phase studies suggest that supplementation with live bacterial products may augment the activity of ICI-based combination therapy in metastatic RCC.

Quavonlimab is a novel CTLA-4 monoclonal antibody currently being explored in combination with pembrolizumab for the treatment of several tumor types.^46,47^ The safety and clinical activity of quavonlimab in combination with an anti-PD-1 antibody have been demonstrated for the treatment of first-line advanced non-small cell lung cancer (NSCLC).^47^ For first-line treatment of advanced ccRCC, there is an ongoing phase 3 study (NCT04736706) investigating the safety and efficacy of coformulated quavonlimab and pembrolizumab plus lenvatinib versus pembrolizumab and lenvatinib.^22^ Botensilimab is a novel Fc-enhanced anti-CTLA-4, shown to enhance T-cell priming while promoting intratumoral Treg depletion. Preclinical and phase 1 studies have demonstrated promising antitumoral activity of botensilimab/balstilimab in “cold tumors” that have been historically unresponsive or resistant to immunotherapy.^48,49^ The phase 2 ARCITECT trial (NCT05928806) is currently examining the combination of botensilimab/balstilimab (anti-CTLA-4/anti-PD1) for first-line treatment of metastatic ccRCC.^50^

#### LAG-3 inhibitor

Lymphocyte-activation gene 3 (LAG-3) is a class of immune checkpoint receptors found on the surface of activated effector T cells and Treg cells and exerts negative regulatory effects on activated immune cells.^51^ LAG-3 and PD-1 are often coexpressed on tumor-infiltrating lymphocytes (TILs), thus contributing to tumor-mediated T-cell exhaustion.^52^ An early clinical study targeting LAG-3 in RCC was a phase 1 trial examining recombinant LAG-3-Ig fusion protein (eftilagimod-α), an agent that agonizes MHC II proteins on antigen presenting cells, enhancing antigen presentation to CD8+ T cells.^53^ While eftilagimod-α induced CD8+ T-cell activation, its antitumor activity in RCC patients was modest, with 7 of 8 patients in the high-dose group and 3 of 11 patients in the low-dose group demonstrating stable disease as their best overall response. Relatlimab, the first-in-class anti-LAG-3 antibody, gained FDA approval for use in combination with nivolumab in first-line unresectable or metastatic melanoma based on the results from the phase 3 RELATIVITY-047 trial.^54^ In RCC, there are ongoing clinical trials examining the efficacy of relatlimab/nivolumab in the context of advanced or metastatic disease (NCT02996110) and in the neoadjuvant setting (NCT05148546). Another anti-LAG3 antibody, favezelimab, coformulated with pembrolizumab is currently under development in phase 1/2 trials for both first-line metastatic RCC (NCT04626479) and pretreated metastatic RCC (NCT04626518). A phase 1/2 basket trial of the LAG-3 inhibitor, ieramilimab (LAG525), in combination with a PD-1 inhibitor, spartalizumab, demonstrated modest antitumor activity, with 13 of the 121 patients achieving a complete or partial response (PR).^55^ A phase 1 study of fianlimab, a LAG-3 inhibitor, plus cemiplimab, an anti-PD-1, for the treatment of ICI-naïve or ICI-pretreated metastatic ccRCC showed an ORR of 20% (3 PRs/15 patients) in the ICI-naïve cohort and 7% (1 PR/15 patients) in the ICI-pretreated cohort.^56^ A summary of trials that are currently investigating anti-LAG-3 antibodies, alone or in combination with a PD-1 inhibitor, are listed in Table 2.

**Table 2.** Select ongoing clinical trials of immunotherapy in advanced RCC.

Treatment	Setting	Phase	Primary endpoints	Identifier
**Immune checkpoint inhibitors**				
Relatlimab (anti-LAG-3)/nivolumab	Neoadjuvant therapy of ccRCC	2	Pathologic response rate	NCT05148546
Botensilimab (anti-CTLA-4) + balstilimab (anti-PD-1) vs Ipilimumab/nivolumab	1L advanced ccRCC	2	ORR	NCT05928806
Pembrolizumab + belzutifan + lenvatinib or Pembrolizumab/quavonlimab (anti-CTLA-4) + lenvatinib vs Pembrolizumab + lenvatinib	1L advanced ccRCC	3	PFS/OS	NCT04736706
Favezelimab (anti-LAG-3)/pembrolizumab + lenvatinib or Vibostolimab (anti-TIGIT)/pembrolizumab + belzutifan vs pembrolizumab + lenvatinib	1L advanced ccRCC	1/2	Lead-in phase: DLT; AEs Efficacy phase: DLT; AEs; ORR	NCT04626479
Relatlimab (anti-LAG-3)/nivolumab	2L + advanced ccRCC after ICI	2	ORR, DOR, PFS	NCT02996110
Pembrolizumab/quavonlimab (anti-CTLA-4) or favezelimab (anti-LAG-3)/pembrolizumab or pembrolizumab + MK-4830 (ILT4 inhibitor)	2L + advanced ccRCC after ICI and TKI	1/2	Lead-in phase: DLT; AEs Efficacy phase: DLT; AEs; ORR	NCT04626518
INCAGN02385 (anti-LAG-3)	Advanced solid tumors with progression after standard treatment	1	AEs	NCT03538028
Tiragolumab (anti-TIGIT) + atezolizumab	Advanced solid tumors with progression after standard treatment	2	ORR	NCT03977467
**CAR-T therapy**				
CAR T-cells targeting CAIX	3L + advanced RCC	1	AEs	NCT04969354
CAR T-cells targeting CD70	Advanced solid tumors with progression after standard treatment	1/2	Phase 1: DLT; AEs Phase 2: ORR	NCT05795595
Allogeneic CAR T-cells targeting CD70	advanced RCC refractory to ICI and TKI therapies	1	DLT; AEs	NCT04696731
**Bispecific antibodies**				
Volrustomig (CTLA-4/PD-1 bispecific Ab) + lenvatinib	1L advanced ccRCC	1	DLT; AEs	NCT04522323
AK112 (PD-1/VEGF bispecific Ab)	Advanced solid tumors with progression after standard treatment	1	DLT; AEs	NCT04047290
XmAb819 (CD3/VEGF bispecific T-cell engager)	advanced RCC refractory to ICI and TKI therapies	1	DLT; AEs	NCT05433142

Abbreviations: 1L, first line; 2L+, second-line or later; 3L+, third-line or later; AEs, adverse events; CAIX, carbonic anhydrase IX; CAR-T, chimeric antigen receptor T cell; ccRCC, clear cell renal cell carcinoma; DLT, dose-limiting toxicity; ICI, immune checkpoint inhibitor; ORR, overall response rate; OS, overall survival; PFS, progression-free survival; TKI, tyrosine kinase inhibitor.

#### TIGIT inhibitor

T-cell immunoglobulin and ITIM domain (TIGIT) is an inhibitory checkpoint receptor that resembles PD-1/PD-L1 mediated signaling in tumor immunity in many tumor types.^57,58^ TIGIT, primarily expressed on T cells and NK cells, has been shown to play a role in CD8+ T-cell exhaustion.^59^ In preclinical models, dual inhibition of TIGIT and PD-L1 or PD-1 synergistically restored effector function in exhausted T cells and augmented the antitumor immune response.^60^ Tiragolumab (anti-TIGIT antibody) in combination with atezolizumab (anti-PD-L1) was investigated for the treatment of PD-L1-positive untreated NSCLC in the phase 2 CITYSCAPE trial.^61^ Patients who received dual TIGIT and PD-L1 blockade demonstrated a longer median PFS compared to patients who received atezolizumab alone. Several early phase clinical trials are currently underway to evaluate the safety and efficacy of TIGIT inhibitors, in combination with an anti-PD-1/PD-L1, for the treatment of metastatic tumors, including RCC (NCT03977467, NCT02913313, NCT04626479). A summary of these studies is provided in Table 2.

### New developments in immunotherapy

#### Chimeric antigen receptor T-cell therapy

The introduction of chimeric antigen receptor (CAR) T-cell therapy has significantly advanced the treatment of hematologic malignancies, providing durable responses in patients with relapsed or refractory disease. Despite the tremendous success of CAR-T therapy in hematologic malignancies, its role in solid malignancies is in early development.^62,63^ The first CAR-T study in RCC targeted the carbonic anhydrase IX (CAIX), a transmembrane protein, that has been found to be upregulated in ccRCC.^64^ The study enrolled 12 patients, with grades II-IV liver toxicities observed in 4 of the 8 patients across the initial 2 cohorts. There was no clinical response observed in this study.^65^ Another CAR-T study targeting VEGF (NCT01218867) was discontinued early following failed futility analysis. An ongoing study of a CAIX-targeted CAR-T regimen in advanced ccRCC involves girentuximab delivery into the hepatic artery prior to CAR-T infusion, aiming to minimize potential hepatotoxicity (NCT04969354).

CD70 is a costimulatory signal expressed on the B and T cells and also found to be highly expressed in ccRCC. The phase 1 COBALT-RCC study evaluated an allogeneic CRISPER-Cas9 engineered anti-CD70 CART-T therapy (CTX130) in patients with relapsed/refractory metastatic ccRCC.^66^ Treatment with CTX130 provided a disease control rate of 81% (13 out of 16 patients), including 12 patients with SD and 1 patient who achieved and remained in CR at 36 months (including at time of study publication).^67^ This case represents the first durable complete response in a patient with RCC to CAR-T therapy. These encouraging results led to the development of CTX131, a modified version of the CTX130 construct with additional knockouts of Regnase-1 (to enhance CAR-T cell persistence) and TGFβR2 (to reduce immunosuppressive activity). Preclinical studies from xenograft RCC models have exhibited improved potency and efficacy with CTX131, which is currently under investigation in a phase 1/2 trial for the treatment of various solid tumors, including metastatic RCC (NCT05795595).

The phase 1 TRAVERSE study (NCT04696731) is currently investigating an anti-CD70 allogenic CAR-T preparation (ALLO-316) for patients with advanced ccRCC who have progressed on ICI and TKI therapies. The initial results reported an ORR of 18% among 17 patients and a disease control rate (DCR) of 82%. Among the 9 patients who were found to have high CD70-expressing tumors, the ORR and DCR were higher at 33% and 100%, respectively.^68^ Several ongoing CAR-T trials are investigating novel target antigens and summarized in Table 2.

#### Bispecific antibodies

Bispecific antibodies (BsAbs) have 2 distinct binding domains that are designed to target 2 different antigens concurrently. XmAb20717 is a bispecific antibody targeting CTLA-4 and PD-1. A phase 1 basket trial, enrolling 77 patients with pretreated advanced solid tumors, reported an ORR of 13%, which included PRs in 2 patients with RCC.^69^ Volrustomig (MEDID5752), a bispecific antibody targeting CTLA-4 and PD-1, was evaluated in an early phase study for untreated advanced ccRCC. The expansion phase randomized patients between a higher (750 mg) and lower dose (500 mg) of volrustomig. Although the higher dose of volrustomig provided a higher DCR compared to the lower dose (90% vs 70%), it was associated with an increased incidence of grade 3 or 4 treatment-related toxicities (63% vs. 42%).^70^ An ongoing trial is examining volrustomig in combination with lenvatinib for first-line treatment of advanced ccRCC (NCT04522323). PM8002, a BsAb targeting PD-L1 and VEGF-A, was investigated in a multitumor phase 1/2 trial of 263 patients with advanced solid tumors. The study reported an ORR of 15.2% (32/211) across all enrolled patients, with a higher ORR of 26.9% (7/26) observed in patients with advanced RCC.^71^

Bispecific T-cell engagers (BiTE) are a class of bispecific antibodies that induce tumor cell killing by simultaneously binding a tumor-associated antigen on one arm and a T-cell associated molecular (most commonly CD3) on the other arm. In solid tumor oncology, early phase trials of BiTE therapy have encountered multiple challenges, including the lack of specific target antigens, tumor heterogeneity, and “on-target, off-tumor” toxicities.^72,73^ However, one promising target antigen to emerge in RCC may be ectonucleotide pyrophosphatase/phosphodiesterase 3 (ENPP3), given its specific expression on RCC cells and minimal expression in healthy tissue. A phase 1 trial is currently examining the safety and preliminary efficacy of XmAb819, a BiTE that targets ENPP3 and CD3, for the treatment of advanced RCC in the refractory setting (NCT05433142). Additional ongoing trials are investigating BsAb and BiTE therapy for the treatment of RCC, with their summaries provided in Table 2.

### Radiopharmaceutical therapy

#### CAIX-targeting agents

The success of prostate-specific membrane antigen (PSMA), labeled with Lutetium-177 (177Lu), in the treatment of patients with castration-resistant prostate cancer^74^ has paved the way for investigating radioligand therapy in the treatment of other tumor types. In ccRCC, a potential tumor target is CAIX, which is overexpressed in more than 90% of ccRCC tumors and has been shown to play a role in disease progression.^75,76^ 177Lu-labeled anti-CA-IX antibodies (177Lu-girentuximab) was previously investigated in a phase 2 study that comprised 14 patients with metastatic ccRCC.^77^ Among the 14 enrolled patients, 8 patients had stable disease and 1 patient achieved a PR after the first cycle. Six patients underwent a second cycle, with 5 patients maintaining stable disease. None of the patients were eligible to receive additional cycles due to prolonged myelotoxicity. Despite these challenges, the initial findings from this study prompted further interest to investigate radiopharmaceuticals for the treatment of advanced RCC. A single-arm phase 2 STARLITE-2 trial is currently evaluating the combination of 177Lu-girentuximab (with a dose escalation lead-in phase) and nivolumab in patients with metastatic ccRCC who progressed after prior IO treatment (NCT05239533).^78^ Additionally, 177Lu-girentuximab in combination with nivolumab and cabozantinib will be investigated in the phase 2 STARLITE-1 trial in patients with untreated advanced ccRCC.^79^ The results from these studies may broaden therapeutic options in the treatment-refractory setting.

A recent small study of 4 patients evaluated the first-in-class CAIX-binding radiolabeled peptide, DPI-4452, conjugated to Gallium-68 (68Ga), as an effective diagnostic imaging method to identify ccRCC lesions.^80^ Administration of 68Ga-DPI-4452 demonstrated intense and sustained radiotracer uptake in RCC tumors with low background activity in normal kidney, liver, and bone marrow tissues, making it a promising diagnostic application for patient selection in future studies on CAIX-directed radioligand therapies. To this end, a phase 1/2 trial examining the safety and antitumor efficacy of 177Lu-DPI-4452 in patients with RCC whose tumors absorb 68Ga-DPI-4452 is currently underway (NCT05706129).

#### PSMA-targeting agents

Prostate-specific membrane antigen (PSMA) is a transmembrane glycoprotein encoded by the *FOLH1* gene that is physiologically expressed not only on prostate cells but also on a variety of extraprostatic tissues, such as lacrimal and salivary glands, kidneys, liver, and bladder.^81^ Approximately 86%-100% of ccRCC tumors have been shown to exhibit positive PSMA expression compared to 0%-28% of papillary RCC.^82^ These findings may support the role of PSMA-based theranostic agents in the management and treatment of ccRCC. PSMA-targeting imaging agents have been investigated for potential use in RCC across several studies, however, a limitation for clinical use in nonmetastatic ccRCC is absorption of PSMA radiotracer in normal kidney parenchyma.^83,84^ One solution may be dynamic 68Ga-PSMA PET acquisition and analysis of radioligand kinetics, which may serve as a valuable tool in distinguishing malignant from benign renal lesions.^85^

The success of PSMA-targeted radioligand therapy in castration resistant prostate cancer has generated interest in extending this approach to the treatment of ccRCC. In a multi-institutional retrospective study of 1765 RCC tumors, the expression of *FOLH1* strongly correlated with angiogenic gene expression in the tumor microenvironment.^86^ Additionally, patients whose tumor had higher *FOLH1* expression tended to have longer treatment duration while on cabozantinib.^86^ These findings may support further studies of PSMA-based theranostics in combination with VEGF-targeted agents in RCC. A multicenter phase 1/2 PRadR study is currently investigating the safety and efficacy of 177Lu-PSMA for patients with metastatic ccRCC who progressed on 2 or more prior lines of therapy (NCT06059014).^87^ A case report of a patient with metastatic RCC treated with 177Lu-PSMA radioligand therapy demonstrated rapid washout of the radionuclide from the tumor, suggesting that PSMA-targeted radioligand formulations may have to be modified to prolong tumor retention for the treatment of RCC.^88^

#### Radium-223

Bone metastases are prevalent in approximately 30% of pts with advanced RCC.^89^ The presence of bone metastases in RCC portend a worse prognosis due to a higher risk of skeletal-related events compared to patients without bone metastases.^90^ Radium-223, an alpha-emitting radioisotope with high affinity for bone, has been shown to prolong survival in men with castration resistant prostate cancer and bone metastases.^91^ A pilot study examined the effects of combining a TKI with Radium-223 for the treatment of metastatic RCC with bone involvement.^92^ This regimen demonstrated safety and led to a decline in markers of bone formation and resorption. Building on these findings, a multi-center phase 2 study was initiated to explore the efficacy of combining Radium-223 with cabozantinib, a VEGF and MET kinase inhibitor with enhanced activity in bone, in patients with metastatic RCC and bone metastases.^93^ This study is currently ongoing (NCT04071223).

### Novel targets and treatment strategies

#### PARP inhibitors

The use of poly (ADP-ribose) polymerase inhibitors (PARPi) has been approved for the treatment of multiple tumor types with DNA damage repair (DDR) alterations.^94^ Multiple clinical trials have evaluated the efficacy of PARPi across genomically defined RCC cohorts. A phase 2 clinical trial of talazoparib, a PARPi, and avelumab, an ICI, demonstrated limited antitumor activity (ORR of 0%, median PFS 3.5 months) in a cohort of 10 patients with *VHL*-altered advanced ccRCC.^95^ Several studies are currently investigating the relevance of PARPi in treating advanced RCC. The phase 2 single-arm ORCHID trial is examining the efficacy of single-agent olaparib in 11 patients with pretreated advanced RCC harboring select DDR alterations who progressed after ICI and/or VEGF TKI (NCT03786796). In an interim analysis, 22% achieved durable disease control, with notable responses demonstrated in the presence of *BAP1* alterations, including one patient with a PR.^96^ In a phase 1 study (NCT03682289), the ataxia telangiectasia and Rad3-related (ATR) kinase inhibitor, ceralasertib, is being examined as a single agent and in combination with olaparib or a PD-L1 inhibitor, durvalumab, across various solid tumor types, including advanced RCC. The results from these studies and others (Table 3) may broaden treatment options for patients with deleterious DDR gene alterations.

**Table 3.** Select ongoing clinical trials of novel strategies in advanced RCC.

Treatment	Setting	Phase	Primary endpoints	Identifier
**TAM receptor TKI**				
PF-07265807 (AXL and Mer receptor TKI) + Sasanlimab (anti-PD-1) + axitinib	Part 3: refractory advanced ccRCC Part 4: 1L advanced ccRCC with IMDC intermediate or poor risk	1	Part 3: DLT; AEs Part 4: ORR	NCT04458259
**PARP inhibitor**				
Multi-arm platform trial: Olaparib (PARPi) monotherapy or cediranib (anti-VEGF-TKI) +/- olaparib or Durvalumab (anti-PD-L1) +/- olaparib	Neoadjuvant therapy of ccRCC	2	Changes in capillary permeability (without anti-PD-L1); changes in intra-tumoral CD8 + T cell infiltration (with anti-PD-L1)	NCT03741426
Olaparib	2L+ advanced RCC with select DDR alterations after ICI and/or TKI	2	ORR	NCT03786796
Ceralasertib (ATR kinase inhibitor) +/- olaparib	2L+ advanced ccRCC	2	ORR	NCT03682289
Niraparib (PARPi) or Dostarlimab (anti-PD-1)	Cohort 3: 2L+ advanced ccRCC with select DDR alterations after anti-PD-1/anti-CTLA-4 doublet or ICI/TKI doublet	2	ORR	NCT04779151
**CDK inhibitor**				
Abemaciclib (CDK4/6 inhibitor) + suntinib	3L+ metastatic ccRCC after Ipilimumab/nivolumab and cabozantinib (IMDC intermediate/poor risk)	1	RP2D	NCT03905889
Part 1: Palbociclib (CDK4/6 inhibitor) + belzutifan Part 2: Belzutifan + palbociclib vs. belzutifan alone	3L+ advanced ccRCC after ICI and TKI	1/2	Phase 1: DLT; AEs Phase 2: ORR	NCT05468697
**Antibody-drug conjugate (ADC)**				
DS-6000a (cadherin 6-directed deruxtecan ADC)	2L+ advanced RCC	1	DLT; AEs	NCT04707248
**Radiopharmaceuticals**				
Lu-177-girentuximab + nivolumab	2L+ advanced ccRCC after ICI	2	MTD; ORR	NCT05239533
Screening method: 68Ga-DPI-4452 Treatment intervention: 177Lu-DPI-4452	Phase 1: 3L+ advanced ccRCC Phase 2: same criteria as phase 1 with CAIX-positive lesions on 68Ga-DPI-4452 imaging	1/2	Phase 1: DLT; AEs Phase 2: ORR	NCT05706129
Lu-177-EB-PSMA-617	treated or untreated RCC	n/a	safety assessed by CTCAE v4	NCT05170555
Radium-223 with cabozantinib	metastatic RCC with ≥2 metastatic bone lesions and ≤ 2 lines of prior treatment	2	Symptomatic skeletal event free survival	NCT04071223
177Lu-PSMA-1	3L + advanced ccRCC after ICI and TKI	1/2	AEs (phase 1); DCR (phase 2)	NCT06059014

Abbreviations: 1L, first line; 2L+, second-line or later; 3L+, third-line or later; AEs, adverse events; CAIX, carbonic anhydrase IX; ccRCC, clear cell renal cell carcinoma; CDK, cyclin-dependent kinase; DCR, disease control rate; DLT, dose-limiting toxicity; ICI, immune checkpoint inhibitor; ORR, overall response rate; OS, overall survival; PARPi, PARP inhibitor; PFS, progression-free survival; TKI, tyrosine kinase inhibitor

#### CDK inhibitors

Cyclin-dependent kinase (CDK) inhibitors have emerged as attractive candidates for RCC treatment due to their ability to target sustained proliferation, regulate the cell-cycle, as well as modulate metabolism, antitumor immunity, and therapeutic resistance.^97,98^ Beyond cell-cycle regulation, CDK4/6 exhibit non-canonical functions in metabolism including insulin signaling and glucose metabolism.^99-101^ This aspect of CDK4/6 activity may be particularly relevant in RCC tumor biology, as dysregulation of metabolic pathways is a fundamental driver of RCC, underscoring the importance of targeting these mechanisms.^102^ Given their potential dual impact on proliferation and metabolism, CDK4/6 inhibitors have emerged as a promising therapeutic option for RCC. Preclinical models with abemaciclib reveal that CDK4/6 can activate mTOR, releasing it from TSC2-mediated inhibition, suggesting that CDK4/6 inhibitors suppress cell proliferation and metabolism via mTOR signaling inhibition.^101^ CDK4/6 inhibitors have also been shown to augment ICI-mediated antitumor activity through various mechanisms, resulting in enhanced tumor regression and OS in several preclinical models.^103-105^ The immunomodulatory properties of CDK4/6 inhibitors, coupled with the clinical success of ICI therapy in RCC, suggest combining these approaches may provide an effective strategy.^106^ A phase 1 study (NCT03905889) is currently investigating the combination of a CDK4/6 inhibitor, abemaciclib, with sunitinib in patients with advanced RCC.

A prior study demonstrated a synthetic lethal relationship between *VHL* inactivation and loss of CDK4/6 activity.^107^ The combination of CDK4/6 inhibitor and belzutifan was shown to promote synergistic suppression of tumor growth ex vivo. These findings support the rationale to leverage this synthetic lethal relationship for the treatment of RCC. The phase 1/2 LITESPARK-024 study is evaluating the safety and efficacy of belzutifan with or without palbociclib, a CDK4/6 inhibitor, in patients with pretreated advanced ccRCC after 2 prior lines of therapy (NCT05468697).

#### Antibody-drug conjugates

The introduction of antibody-drug conjugate (ADC) therapy has advanced the treatment of various solid tumors types in the frontline and pretreated settings. Although several ADCs have been investigated for the treatment of RCC, the clinical efficacy has largely been modest. As previously discussed in this article, ENPP3 is highly expressed in ccRCC, making it a potential target in the development of ADCs.^108^ Two anti-ENPP3 targeting ADCs (AGS-16M8F, AGS-16C3F) were investigated in 2 phase 1 studies. AGS-16M8F study closed without reaching the maximal tolerated dose.^109^ AGS-16C3F entered the phase 2 study in pretreated RCC, but did not meet the primary PFS endpoint.^110^ Another potential target may be cadherin 6 (CDH6), a cell-adhesion molecule involved in epithelial-mesenchymal transition, given its high expression in RCC.^111,112^ DS-6000a, an antibody-drug conjugate composed of an anti-CDH6 monoclonal antibody attached to a deruxtecan payload, is under investigation in a phase I study of pretreated ovarian cancer and advanced RCC (NCT04707248).^113^

## Conclusions

The treatment landscape of metastatic RCC has markedly expanded since the approval of IL-2 and interferon-alpha in the 1990s. Since the introduction of dual ICI or ICI/TKI combination regimens for the treatment of metastatic RCC, multiple first-line immunotherapy-based regimens have become available for patients with metastatic RCC. Unfortunately, there remains an unmet need for patients who do not initially respond or eventually develop resistance to frontline ICI-based combination therapy. Accordingly, there are tremendous efforts and ongoing trials to broaden therapeutic targets and treatment options in the setting of treatment-refractory metastatic RCC. This article summarizes therapies that are currently under development including TKI-targeting novel receptors, radioligand theranostic agents, HIF-2α inhibitors, PARP inhibitors, antibody-drug conjugates, CAR-T, and immune checkpoint inhibitors. The development of safe and effective novel treatment options, along with a deeper understanding of the tumor biology of RCC, may provide durable responses and possibility of cure, for patients with advanced RCC who do not respond to current standards of care.

## Data Availability

No new data were generated or analyzed in support of this research.
